# Hybrid Breeding for Restoration of Threatened Forest Trees: Evidence for Incorporating Disease Tolerance in *Juglans cinerea*

**DOI:** 10.3389/fpls.2020.580693

**Published:** 2020-10-16

**Authors:** Andrea N. Brennan, James R. McKenna, Sean M. Hoban, Douglass F. Jacobs

**Affiliations:** ^1^Department of Forestry and Natural Resources, Purdue University, West Lafayette, IN, United States; ^2^USDA Forest Service, Northern Research Station, West Lafayette, IN, United States; ^3^The Morton Arboretum, Lisle, IL, United States

**Keywords:** butternut, *Ophiognomonia clavigignenti-juglandacearum*, fungal disease, inoculation, resistance breeding, Japanese walnut, conservation

## Abstract

Hybridization is a potential tool for incorporating stress tolerance in plants, particularly to pests and diseases, in support of restoration and conservation efforts. Butternut (*Juglans cinerea*) is a species for which hybridization has only recently begun being explored. This North American hardwood tree is threatened due to *Ophiognomonia clavigignenti-juglandacearum* (*Ocj*), the causal fungus of butternut canker disease (BCD), first observed in 1967. Observational evidence in some wild *J. cinerea* populations indicates that naturalized hybrids of *J. cinerea* with Japanese walnut (*Juglans ailantifolia*) may be more tolerant to BCD than non-admixed *J. cinerea*, but this has not been formally tested in a controlled trial. We aimed to examine potential BCD tolerance within and between *J. cinerea* and *J. cinerea* × *J. ailantifolia* hybrids and to determine if there is a difference in canker growth between BCD fungal isolates. Five-year-old *J. cinerea* and hybrid trees were inoculated with two *Ocj* fungal isolates collected from natural infections found in two different sites in Indiana, United States, and a blank control (agar only). Measurements of both artificially induced and naturally occurring cankers were taken at 8-, 12-, 20-, 24-, and 32-month post-inoculation. Differences in canker presence/absence and size were observed by fungal isolate, which could help explain some of the differences in BCD severity seen between *J. cinerea* populations. Smaller and fewer cankers and greater genetic gains were seen in hybrid families, demonstrating that hybrids warrant further evaluation as a possible breeding tool for developing BCD-resistant *J. cinerea* trees.

## Introduction

Native and non-native diseases and pests are increasingly threatening ecosystems, especially forests, across the globe (Ennos, 2015; Early et al., 2016). This is driven in large part by anthropogenically driven activities, such as globalization and mass trade of plant material that inadvertently transports new pests and pathogens into novel environments (Early et al., 2016). Climate change compounds the problem by providing ideal environments for pests and pathogens (Dukes et al., 2009) and weakening host species, making the host species more vulnerable to attack (Diez et al., 2012). Some species are not able to acclimate or adapt to these increased threats and are facing extinction (Thomas et al., 2004; Bellard et al., 2016).

Hybridization is currently under consideration as a possible tool to incorporate stress tolerance in support of restoration and conservation efforts (Hamilton and Miller, 2015). There are concerns that hybrids could be detrimental to both the target species and its ecosystem through potential invasion (Muhlfeld et al., 2014), outbreeding depression (genetic incompatibilities or reduced fitness; Allendorf et al., 2013), and genetic swamping (loss of local adaptations by genetic dominance from another species; Allendorf et al., 2013). However, desirable traits, such as disease and pest resistance conferred through hybridization, may be one of few remaining tools to save some species (Sniezko and Koch, 2017). Perhaps, the most notable example of using hybridization to support an endangered species is the American chestnut [*Castanea dentata* (Marsh.) Borkh.], which has been crossed with the Chinese chestnut (*Castanea mollissima* Blume) in pursuit of resistance to chestnut blight [*Cryphonectria parasitica* (Murrill) Barr.; Steiner et al., 2017; Clark et al., 2019; TACF, 2020]. The American Chestnut Foundation (TACF), one of the leading organizations in this effort, has been breeding and backcrossing *C. dentata* hybrids for three generations over 30 years and is currently trialing hybrids with increased resistance in several restoration sites in the eastern United States (TACF, 2020).

Another, lesser-known example where hybridization is being considered to save an endangered plant species, is butternut (*Juglans cinerea* L.), a North American hardwood tree species (Michler et al., 2006). While *J. cinerea* shares a native range in the eastern United States similar to black walnut (*Juglans nigra* L.), *J. cinerea* does not extend as far south and is one of few deciduous tree species in the far northern areas of the United States and southern Canada (Rink, 1990; Farrar, 2017). As a masting species, the tree is ecologically important for providing large, energy-rich nuts for both wildlife and humans (Schultz, 2003), but also holds economic importance through high quality wood products (Forest Products Laboratory, 2010). Culturally, *J. cinerea* has been used by Native Americans for a wide variety of purposes, including for medicine, food, dyes, and canoe construction (Moerman, 1998). Medicinally, *J. cinerea* has been documented to have a broader spectrum of antimicrobial activity compared to many other North American hardwood species (Omar et al., 2000).

Unfortunately, *J. cinerea* populations are now in severe decline due to butternut canker disease (BCD), caused by the fungus *Ophiognomonia clavigignenti-juglandacearum* (*Ocj*; Nair, Kostichka, & Kuntz; Broders and Boland, 2011). The disease, first reported in Wisconsin in 1967 (Relund, 1971), manifests as vertically oriented, elliptical cankers that develop on limbs and boles, often causing the surrounding outer bark to peel (Tisserat and Kuntz, 1984). Over time, the cankers multiply and coalesce, ultimately girdling and killing affected trees (Tisserat and Kuntz, 1984). The reduction in *J. cinerea* populations by BCD has nearly eliminated natural regeneration (Boraks and Broders, 2014), to the point that it is now considered endangered by the International Union for Conservation of Nature (Stritch and Barstow, 2019). *Juglans cinerea* is also listed under Canada’s Species at Risk Act (Environment Canada, 2010) and in the United States; the species has a conservation status of either critically imperiled (S1), imperiled (S2), or vulnerable (S3) in 21 states (NatureServe, 2017).

Despite the sporadic occurrence of healthy *J. cinerea* trees in the wild, no durable resistance to BCD has been found in populations of *J. cinerea* to date, with all showing susceptibility upon further testing. For example, when Ostry and Moore (2008) inoculated grafted clones from 12 canker-free source trees with *Ocj*, all individuals displayed susceptibility to the disease. This has led to the concept of using hybridization to incorporate disease resistance into the species (Michler et al., 2006; McKenna et al., 2011; Boraks and Broders, 2014). *Juglans cinerea* does not hybridize with *J. nigra*, the only other *Juglans* conspecific in the eastern deciduous forest (Rink, 1990). However, *J. cinerea* does hybridize with the Japanese walnut (*Juglans ailantifolia* Carr.; Rink, 1990). A study of wild populations of both non-admixed *J. cinerea* and its naturalized hybrids with *J. ailantifolia* found possible tolerance in hybrids compared to its native progenitor, with *J. cinerea* exhibiting an average of 4.5 cankers per tree vs. an average of 2.5 for its hybrids (Boraks and Broders, 2014). However, there have been no controlled evaluations to formally test whether the hybrids hold increased BCD tolerance to *J. cinerea*.

The objectives of this study were to examine potential BCD tolerance within and between non-admixed *J. cinerea* (“*J. cinerea*”) and *J. cinerea* × *J. ailantifolia* hybrids (“hybrids,” unless otherwise noted) and to determine if there is a difference in canker growth between isolates of *Ocj*. Our hypotheses were as follows: (1) hybrids will have greater tolerance to BCD than *J. cinerea*; (2) some *J. cinerea* and hybrid families will show greater tolerance to BCD than other families; and (3) there will be a difference in canker infection by different *Ocj* isolates. To test these hypotheses, a multi-year field study was conducted using *J. cinerea*, and hybrid trees inoculated with two different isolates of *Ocj*.

## Materials and Methods

### Plant Material

In the fall of 2002, seeds were collected from presumed *J. cinerea* trees in an open-pollinated clone bank in Rosemount, MN, United States originating from putatively resistant surviving trees in the wild (family accessions 709–750; Table 1; Supplementary Table S1). Seeds were also collected from six wild presumed *J. cinerea* trees in northern Indiana, United States (family accessions 702–708). The seeds were stratified in a cooler at 2.8°C through winter and germinated in a greenhouse in April 2003. The sprouted seeds were planted in a lowland field of Purdue University’s Martell Forest (West Lafayette, IN, United States 40.4313991, −87.0389821) in May 2003. Approximately, 10 seedlings were planted per family (half-sib progenies sharing the same maternal parent) as two five-tree plots in a randomized row-block design with a spacing of 3.7 m between rows and 1.8 m within rows.

**Table 1:** Best Linear Unbiased Predictors (BLUPs), accuracy estimates, breeding values (BVs), and genetic gains of families of *Juglans cinerea* and its hybrids with *Juglans ailantifolia* based on canker size (area).

Family	Species/hybrid	BLUP	Accuracy	BV	Gain (%)
707	Hybrid	−0.57	0.75	7.10	14
706	Hybrid	−0.46	0.67	7.32	11
711	Hybrid	−0.43	0.84	7.37	11
750	Hybrid	−0.37	0.81	7.49	9
704	Hybrid	−0.35	0.83	7.53	9
702	Hybrid	−0.34	0.81	7.57	8
748	Hybrid	−0.28	0.78	7.69	7
736	*Juglans cinerea*	−0.20	0.85	7.84	5
712	*Juglan cinerea*	−0.19	0.80	7.86	5
710	Hybrid	−0.12	0.83	8.00	3
713	*Juglan cinerea*	−0.09	0.76	8.06	2
717	*Juglan cinerea*	−0.08	0.81	8.07	2
730	*Juglan cinerea*	−0.08	0.85	8.07	2
709	*Juglan cinerea*	−0.08	0.86	8.08	2
731	Hybrid	−0.05	0.83	8.13	1
738	*Juglan cinerea*	−0.03	0.80	8.18	1
734	Hybrid	0.01	0.80	8.26	0
714	*Juglan cinerea*	0.03	0.84	8.29	−1
742	*Juglan cinerea*	0.06	0.80	8.36	−1
708	Hybrid	0.07	0.80	8.38	−2
728	*Juglan cinerea*	0.08	0.81	8.39	−2
722	*Juglan cinerea*	0.09	0.86	8.41	−2
732	Hybrid	0.09	0.81	8.42	−2
727	*Juglan cinerea*	0.12	0.83	8.48	−3
715	*Juglan cinerea*	0.13	0.76	8.50	−3
723	*Juglan cinerea*	0.19	0.84	8.61	−5
747	*Juglan cinerea*	0.20	0.86	8.63	−5
726	*Juglan cinerea*	0.20	0.80	8.63	−5
743	*Juglan cinerea*	0.21	0.84	8.65	−5
733	*Juglan cinerea*	0.22	0.85	8.67	−5
744	*Juglan cinerea*	0.26	0.82	8.75	−6
741	*Juglan cinerea*	0.27	0.81	8.78	−7
718	*Juglan cinerea*	0.33	0.87	8.90	−8
746	*Juglan cinerea*	0.33	0.85	8.90	−8
735	Hybrid	0.36	0.86	8.95	−9
716	*Juglan cinerea*	0.49	0.81	9.22	−12

Cankers were measured 32 months following inoculation with *Ophiognomonia clavigignenti-juglandacearum* (*Ocj*), the causal fungus of butternut canker disease (BCD). A positive genetic gain indicates a family with canker sizes smaller than the population mean, while a negative genetic gain indicates a family with canker sizes greater than the population mean. Population mean = 8.237 (log transformed from mm^2^).

Family variance = 0.098.

An initial visual screening of the seeds was conducted to exclude F1 hybrids at planting. Our original goal was to include only *J. cinerea* families and in particular, those from healthy wild trees that we considered as putatively resistant parents. However, by the third growing season in 2005, early genetic identification methods were being developed (Aradhya et al., 2006; Zhao and Woeste, 2011), and many *J. cinerea* × *J. ailantifolia* hybrids among our *J. cinerea* germplasm collection had been detected which allowed us to examine these “complex” hybrids for phenotypic differences in leaf size, twig color, and terminal and lateral bud characteristics to distinguish these from *J. cinerea*. For the families in the present study, the phenotypic traits of seedlings were rated in the fall of 2005 by two independent observers as 2 = *J. cinerea*, 1 = *J. cinerea* and hybrid mix, or 0 = hybrid, using the methods that ultimately became the basis for those of Woeste et al. (2009). We recognize that phenotypic assessment is imperfect, but Hoban and Romero-Severson (2011) found that nut growers only using their own personal experience and no key, were able to correctly identify their *J. cinerea* or hybrid trees 85% of the time. Therefore, we have high confidence that phenotypic methods used by expert foresters with long experience with these species should be able to make successful species designations in most cases. However, we also performed DNA tests on a subset of individuals from all families in 2009 using chloroplast markers (Aradhya et al., 2006; Zhao and Woeste, 2011), as well as ITS region, mitochondrial, and nuclear markers (Zhao and Woeste, 2011), which confirmed the initial phenotypic *J. cinerea* or hybrid genotype of each family. Further, a second subsample of 39 *J. cinerea* and hybrid trees from those included in the current study were also genetically analyzed in 2019 using the nuclear markers of Hoban et al. (2008) and chloroplast markers of McCleary et al. (2009). For the 31 samples that successfully amplified, the results of this genetic analysis subsample matched with the initial identification designations. From these analyses, we determined that seven of the Rosemount families and all six wild-collected Indiana families were *J. cinerea* × *J. ailantifolia* hybrids. Ultimately, 203 *J. cinerea* trees from 23 different families and 106 hybrid trees from 13 different families were included in the study.

### Inoculations

Two different fungal isolates of *Ocj* were used for the inoculations. Both were collected from natural, spontaneous infections found in Indiana, the first from one of our seedlings in a breeding block at Martell Forest in West Lafayette (IN-1375-4A, “isolate 1”) and the second from the Hoosier National Forest in southern Indiana (IN-1378-3, “isolate 2”). These were chosen in order to use isolates representative of the state in which the study was being conducted, and these specific isolates had already been collected and isolated by Michael Ostry and Melanie Moore (USDA Forest Service, Northern Research Station, St. Paul, MN, United States) and thus were readily available. Samples for initiating cultures were collected from cankered branches in early August 2008 and grown on malt agar in darkness at 20°C. Inoculum was prepared from sporulating cultures after 2 months. Inoculations were applied to the trees at 5 years old in 2008, from late September to early October, when trees have been shown to be most susceptible to infection from *Ocj* (Ostry and Moore, 2008). The inoculation application method was similar to that developed by Anagnostakis (1992) for screening chestnut trees (*Castanea* spp.) for tolerance to chestnut blight. Holes (6-mm diameter) were drilled into the main trunk at approximately breast height, through the bark and slightly into the sapwood. A 6-mm diameter plug of inoculum (agar with *Ocj*) was then inserted into each hole, with fungal hyphae facing inward, toward the cambium. A single layer of masking tape was then wrapped around each inoculation wound. Each hole was spaced 20 cm apart, running in a vertical line down the trunk. Each tree received five inoculation points in the following order: the first, top-most (apical) hole was plugged with a blank control (agar only); the second and third holes with *Ocj* isolate 1; and the fourth and fifth holes with *Ocj* isolate 2.

### Evaluation

Survival was recorded each time canker growth was measured. Cankers resulting from the inoculations were evaluated at 8, 12, 20, 24, and 32 months after the inoculations were applied. The maximum vertical lengths (*l*) and horizontal widths (*w*) of each canker were recorded. The canker length and width were used to calculate the area (*A*) of the inoculated canker, using the formula for an ellipse (oval):

(1) $$A=\frac{l}{2}\times \frac{w}{2}\times \pi \left({\text{cm}}^{2}\right)$$

Cankers occurring from natural *Ocj* infection (outside of inoculation areas) began appearing 4 years after planting in 2006, which was confirmed by isolation of the fungus from several samples of the naturally formed cankers in August 2008. Evaluations of the natural cankers were conducted concurrently with the artificially induced cankers at 8, 20, and 32 months following the inoculations. Natural cankers were rated for cumulative incidence and size using an ordinal scale. Incidence was rated from 0 to 3, where 0 = no natural cankers; 1 = 1 or 2 cankers; 2 = 3–5 cankers; and 3 = 6 or more cankers (McKenna et al., 2011). Size was based on the average size of the natural cankers (length × width), rated from 0 to 3, where 0 (none to very small) = less than ~30 × 10 mm; 1 (small) = ~30–59 × 10–19 mm; 2 (medium) = ~60–99 × 20–24 mm; and 3 (large) = ~100 × 25 mm or greater sized cankers (McKenna et al., 2011).

### Data Analysis

All data was analyzed in R v. 3.5.3 (R Core Team, 2019). There was insufficient mortality by the conclusion of the study to conduct a valid statistical analysis of survival, so only survival percentages are reported. The control inoculations did not produce cankers and were not included in the statistical analyses. Canker growth for the remaining inoculations was analyzed at the species/hybrid level using a two-part model to account for the high level of zero growth instances in the early time points of the study. Both parts of the model were conducted using R package “lme4” (Bates et al., 2015). The first part used a linear mixed model to analyze the percent of individuals in each family with canker growth present over time. The second part evaluated canker area over time with linear mixed models only for inoculations where growth was present, using natural-log-transformed data to meet the assumption of normality of errors. For both parts, species/hybrid, fungal isolate, time, and block within the plot (three-level categorical variable) were considered fixed effects, and family was considered a random effect. Since the second part of the model evaluated at the individual level, individual tree was also included as random and nested within family. To facilitate breeding selection and evaluate variation at the family level, Best Linear Unbiased Predictors (BLUPs; Isik et al., 2017) were generated from a linear mixed model, as in the second part of the inoculated canker model. However, only a subset of the data was used to analyze canker area for inoculations where growth was present at the last time point (32-month post-inoculation), thus, time was not included in the analysis of this data subset. The BLUPs (random effects) for each family were taken from the model and estimates of accuracy were calculated for each BLUP based on its SE and the family variance (S) as (Mrode, 2014):

(2) $$Accuracy=\sqrt{1-\left(\frac{SE^{2}}{S}\right)}$$

Accuracy estimates are the correlation between true and predicted breeding values (BVs; Mrode, 2014) and are used in plant and animal breeding to evaluate confidence in predictions in lieu of the SE (Isik et al., 2017). The BLUPs were converted to BV by multiplying by two and adding the 32-month canker area population grand mean (*μ*). The BV was then converted to a percent gain relative to the population mean:

(3) $$Genetic\;gain=\frac{\mu -BV}{\mu }\times 100\;(\%)$$

A positive genetic gain indicates a family with artificial canker sizes smaller than the population mean, while a negative genetic gain indicates a family with canker sizes greater than the population mean. The families were finally ranked in order of greatest to smallest gains to assist in family breeding selection. The incidence and size of naturally formed cankers were analyzed using cumulative link mixed models (also called ordinal regression or proportional odds models) with R package “ordinal” (Christensen, 2019). Species/hybrid, fungal isolate, time, and plot block were set as fixed effects. Individual tree nested within family were set as random effects.

## Results

### Survival

By the conclusion of the study (32-month post-inoculation), there was 96 and 92% survival for *J. cinerea* and hybrid trees, respectively.

### Artificially Induced Infection

**Figure 1:**
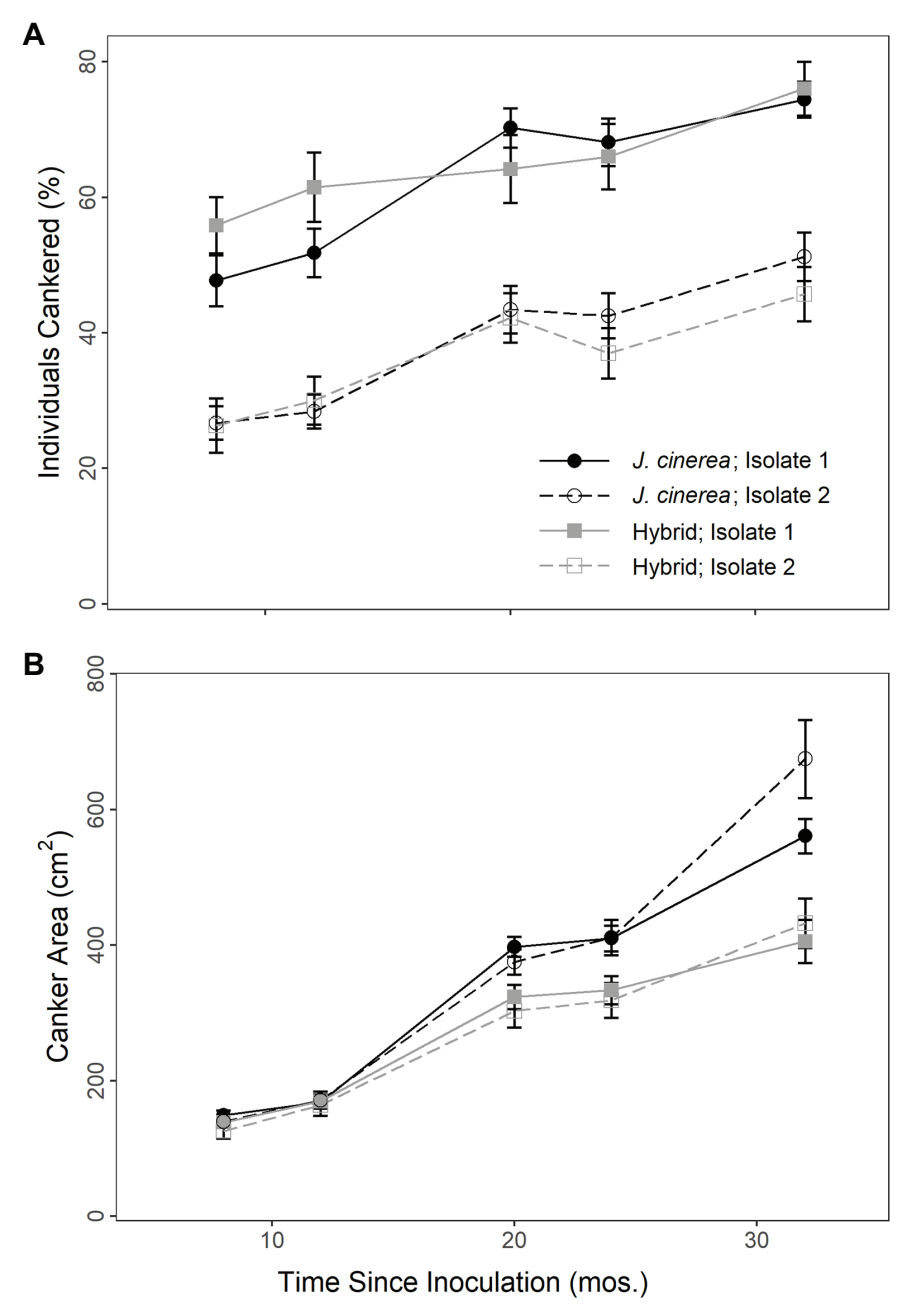
Percent of individuals cankered **(A)** and canker area **(B)** over time on *J. cinerea* and its hybrids with *J. ailantifolia* following inoculation with two different isolates of *Ocj*, the causal fungus of butternut canker disease. Isolate significantly affected both percent of individuals cankered (*p* < 0.0001) and canker area (*p* = 0.021). Species/hybrid affected canker area (*p* = 0.003), but not percent of individuals cankered (*p* = 0.713).

The percent of individuals with canker growth present at the inoculation site strongly increased over time (*χ*^2^ = 186.87, *p* < 0.0001; Figure 1A). There was no difference in the presence of canker growth following inoculation between *J. cinerea* and hybrid trees (*χ*^2^ = 0.14, *p* = 0.713). However, there was a strong difference by fungal isolate (*χ*^2^ = 421.48, *p* < 0.0001), with much greater presence of canker growth resulting from inoculations with isolate 1 than isolate 2. There was a moderate interaction between species and time (*χ*^2^ = 5.74, *p* = 0.017), but there was no interaction evident between species/hybrid and fungal isolate (*χ*^2^ = 2.94, *p* = 0.086); fungal isolate and time (*χ*^2^ = 0.13, *p* = 0.720); or species/hybrid, fungal isolate, and time (*χ*^2^ = 0.20, *p* = 0.657).

The size of cankers resulting from the inoculations strongly increased over time (*χ*^2^ = 1418.95, *p* < 0.0001; Figure 1B). Canker growth on the hybrids was smaller than on *J. cinerea* and by the final timepoint, the average inoculated canker area (non-zero) on hybrid trees was 41.9 (± 3.4) cm^2^ compared to 61.8 (± 4.1) cm^2^ on *J. cinerea* trees (*χ*^2^ = 8.65, *p* = 0.003). There was also a difference in fungal isolate, with an average canker area of 48.3 (± 2.9) cm^2^ for isolate 1 vs. 55.4 (± 4.7) cm^2^ for isolate 2 by the final timepoint (*χ*^2^ = 5.34, *p* = 0.021). A strong interaction was present between species/hybrid and time (*χ*^2^ = 19.78, *p* < 0.0001), with canker growth increasing more rapidly in *J. cinerea* than the hybrids. However, there was no interaction evident between species/hybrid and fungal isolate (*χ*^2^ = 0.31, *p* = 0.580); fungal isolate and time (*χ*^2^ = 0.54, *p* = 0.463); or species/hybrid, fungal isolate, and time (*χ*^2^ = 0.97, *p* = 0.325).

By the conclusion of the study at 32-month post-inoculation, genetic gains based on canker size ranged from −12 to 14% (Table 1). There was distinct separation of families by genetic gains based on canker size. In the top-ranking quarter (5–14% gains), seven of nine families were hybrids, while in the bottom quarter (−12 to −5% gains), eight of nine of families were *J. cinerea*.

### Naturally Occurring Infection

**Figure 2:**
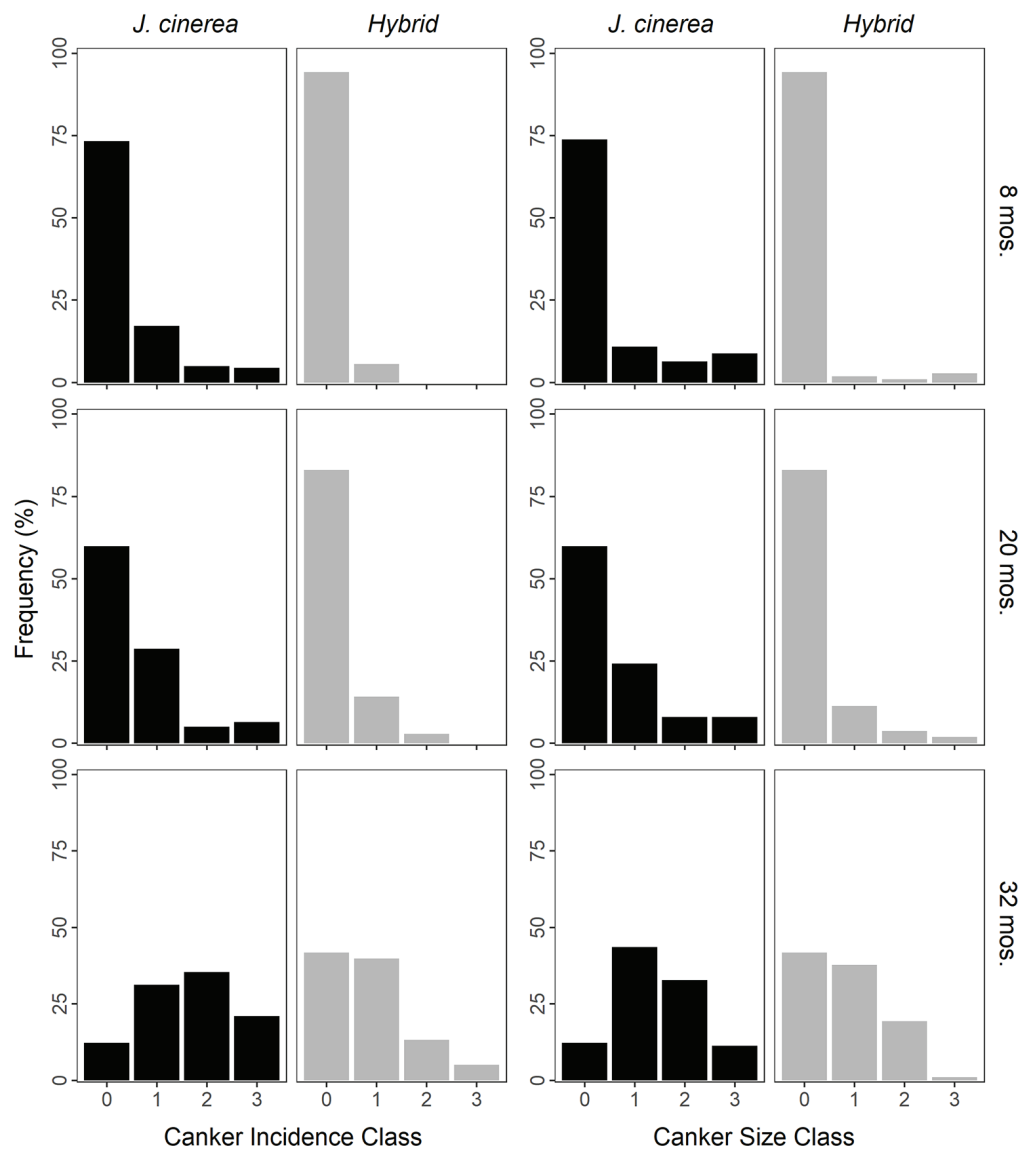
Frequency of trees of *J. cinerea* and its hybrids with *J. ailantifolia* with naturally occurring cankers by different incidence and size classes over time since the initiation of the study. Cankers were formed by *Ocj*, the causal fungus of butternut canker disease. Incidence was rated from classes 0 (no natural cankers) up to 3 (6 or more cankers). Size was based on the average size of the natural cankers (length × width), rated from classes 0 (none to very small; less than ~30 × 10 mm) up to 3 (large; ~100 × 25 mm or greater). *Juglans cinerea* and hybrids were significantly different for both natural canker incidence and size at all timepoints (*p* < 0.0001 for all).

Incidence of naturally occurring cankers increased strongly over time (*χ*^2^ = 404.76, *p* < 0.0001; Figure 2). Species/hybrid was also an important predictor of natural canker incidence, with *J. cinerea* having a greater incidence of natural cankers than the hybrids at all timepoints (*χ*^2^ = 24.53, *p* < 0.0001). As an example, by the final timepoint, 12 and 21% of *J. cinerea* had natural cankers in classes 0 (lowest incidence) and 3 (greatest incidence), respectively, compared to 42 and 5% in hybrids (Figure 2). There was no evidence of an interaction between species/hybrid and time for natural canker incidence (*χ*^2^ = 2.67, *p* = 0.263).

The size of naturally occurring cankers increased greatly over time (*χ*^2^ = 264.82, *p* < 0.0001; Figure 2). *Juglans cinerea* had larger natural cankers than the hybrids at all timepoints (*χ*^2^ = 23.95, *p* < 0.0001). At the final timepoint, 12 and 11% of *J. cinerea* had cankers in size classes 0 (smallest) and 3 (largest), respectively, vs. 42 and 1% of hybrids (Figure 2). No evidence of an interaction between species/hybrid and time was found for the size of natural cankers (*χ*^2^ = 2.62, *p* = 0.270).

## Discussion

### Effect of Fungal Isolate

Supporting our hypothesis, the two *Ocj* isolates used for the inoculations in our study resulted in different levels of canker occurrence and size, which is consistent with studies by Ostry and Moore (2008) and Broders et al. (2012, 2015). In the current study, although the specific fungal isolate used in inoculations played a role in canker size, isolate played a much larger role in predicting the presence/absence of canker growth. This could indicate stronger variability in the ability of different *Ocj* isolates to initiate host infection. With differing levels of aggressiveness, the specific isolates present within a certain location may contribute, in part, to help explain why some areas experience more severe and sudden BCD outbreaks than others (Broders et al., 2015; Morin et al., 2017). However, it is likely that habitat and environment also play a strong role in determining occurrence of infection in these situations as well (Boraks and Broders, 2014; Labonte et al., 2015; Morin et al., 2017).

### Tolerance of *Juglans cinerea* and Its Hybrids

Although there was no significant difference in inoculated canker absence/presence between *J. cinerea* and its hybrids, the hybrids did have smaller cankers (averaging nearly 1/3 smaller by the end of the study) that grew slower than those on the progenitor species. Further, hybrid families had the greatest genetic gains in terms of canker size by 32-month post-inoculation. When considering naturally occurring infection, the hybrids also had both fewer and smaller cankers than *J. cinerea*. Thus, our hypotheses that hybrids would show greater tolerance to BCD than *J. cinerea* was mostly supported. This trend was also seen in a study of populations of wild *J. cinerea* and naturalized hybrids in the northeastern United States, where the hybrids were found to be much less affected by the disease and had fewer cankers, less dieback, and greater vigor than trees of *J. cinerea* (Boraks and Broders, 2014). It should be noted, however, that while hybrids in the current study were more tolerant on average than *J. cinerea*, some hybrids performed worse than average and some *J. cinerea* performed better than average (Table 1).

Black and Neely (1978) reported that *J. cinerea* × *J. ailantifolia* hybrids also had greater tolerance than *J. cinerea* to another *Ophiognomonia* fungal species, anthracnose [*Ophiognomonia leptostyla* (Fr.) Sognov]. These results in *J. cinerea* can be compared to hybrids and other diseases in *Juglans*. In the aforementioned study, hybrids of *J. nigra* with four other *Juglans* species consistently showed greater anthracnose tolerance than their highly susceptible *J. nigra* parent (Black and Neely, 1978). Conversely, in another study, hybrids of Persian walnut (*Juglans regia* L.) and iron walnut (*Juglans sigllata* Dode) showed similar or even greater susceptibility to walnut bacterial blight (*Xanthomonas arboricola* pv. *juglandis* Pierce) than both their progenitors (Jiang et al., 2019). Heightened susceptibility to crown gall disease (*Agrobacterium tumefaciens* Smith & Townsend) has also been documented in hybrids of northern California black walnut [*Juglans hindsii* (Jeps.) Jeps. ex R.E. Sm.] and *J. regia* (McKenna and Epstein, 2003). Thus, disease tolerance in *Juglans* hybrids that is greater than one or both of the parents is not guaranteed and depends on the specific host-pathogen interaction for each disease. Further, in relation to pest resistance, *J. ailantifolia* and its hybrids with both *J. cinerea* and *J. nigra* have expressed greater susceptibility to butternut curculio (*Conotrachelus juglandis* LeConte) than the two native North American progenitors (Wilson and Corneil, 1978). This illustrates that in attempting to obtain BCD resistance in *J. cinerea*, it will be critical that increased susceptibility to native pests not also be inadvertently incorporated.

Interspecific hybrids have also been developed in other genera with the goal of incorporating disease resistance or tolerance into a susceptible and endangered native species. As discussed previously, *C. dentata* × *C. mollissima* hybrids backcrossed to *C. dentata* have been developed with increased resistance to chestnut blight compared to their susceptible *C. dentata* progenitor (Steiner et al., 2017; Clark et al., 2019; TACF, 2020). After strong selection for *C. dentata*-specific traits and blight resistance, second (B_2_) and third (B_3_) backcross hybrid lines developed at TACF’s Meadowview Research Farm (Meadowview, VA, United States) were found to have average blight areas (B_2_) or blight ratings (B_3_), significantly different and intermediate to their American and Chinese chestnut progenitors, but not different from those of the F_1_ generation (Steiner et al., 2017). However, Clark et al. (2019) reported that blight resistance in *C. dentata*, *C. mollissima*, B_1_, B_2_, and B_3_ Meadowview backcross hybrids ultimately varied when planted across different sites in the first natural forest field trials testing this resistance. While the *Castanea* hybrids held yearly resistance rankings that were intermediate to that of their progenitors in two of the sites (NC and VA), there was no significant difference between any of the progenitors or hybrids in a third site (TN). Given such genotype × environment variation, it is essential that future work test *J. cinerea* and hybrid families in common garden plots across multiple sites in order to assess the durability of possible BCD resistance. Efforts have also been pursued to develop Dutch elm disease (*Ophopstoma* spp.) resistant hybrids for restoring the endangered American elm (*Ulmus americana* L.) and several other *Ulmus* spp. affected by the disease (Brunet and Guries, 2016; Griffin et al., 2017; Martín et al., 2019). While progress has been made with promising hybrids and a few *U. americana* varieties (Brunet and Guries, 2016; Griffin et al., 2017; Martín et al., 2019), it has been slowed by incompatibility and ploidy barriers (Ager and Guries, 1982). These issues do not appear to be an issue with *J. cinerea* × *J. ailantifolia* hybrids given the large number of naturalized hybrids present in the landscape (Hoban et al., 2009).

Consistent with our second hypothesis, both *J. cinerea* and hybrid families separated out by genetic gains on 32-month canker size, with some families showing greater tolerance than others, indicating a possible genetic basis to disease tolerance (Isik et al., 2017). While hybrids tended to rank highest in genetic gains, some *J. cinerea* families, such as 736 and 712, had modest gains as well. However, the finding of a potential genetic basis to BCD tolerance in the current research must be compared to a heritability study of a wild population of *J. cinerea* in Wisconsin. Labonte et al. (2015) primarily concluded that genetic differences explained little of the variance in mortality, and that environmental and site differences were stronger predictors. It was also reported that while genetics was not correlated with survival, there were low, but significant correlations between genetics and canker-related traits, including canker number, which is consistent with the present study. The population assessed by Labonte et al. (2015) only contained non-admixed *J. cinerea* trees, which are believed to have originated from a small number of mother trees, limiting the genetic diversity. The present study, in contrast, included seeds propagated from long-term surviving selections collected from across a wide geographic range and inter-pollinated together in a grafted orchard, expanding the genetic diversity of our test families. Additionally, our study did not include environmental and site factors as in Labonte et al. (2015), so a comparison with the current study’s results on heritability of BCD tolerance in hybrids is not entirely possible.

Survival, as assessed by Labonte et al. (2015), is likely a better measure in ultimately identifying the most BCD tolerant trees than the canker-related traits we evaluated in just under 3 years. However, the high survival (over 90%) for both *J. cinerea* and hybrid trees by the conclusion of the present study suggests that more than 32 months are required to understand the full potential of tolerance differences between the species and hybrids once *Ocj* infection begins. Further, the research of Labonte (2015), as well as Clark et al. (2019) with *C. dentata* (discussed earlier), both underscore the need for BCD tolerance screenings on multiple different sites to understand possible genotype × environment interactions. Sambaraju et al. (2018) reported that multiple factors, notably weather, influence *Ocj* epidemiology. It is likely that the successful restoration of *J. cinerea* will not be accomplished solely through the integration of genetic BCD resistance, but in combination with appropriate site selection and silvicultural practices (Jacobs et al., 2013).

Ultimately, beyond any increased disease tolerance or resistance that hybrids may hold compared to their progenitor species, it is essential to also consider how well such hybrids fill both the economic and ecological niches of the progenitor species they are intended to replace, including reproductive potential, physiology, invasiveness, and wood quality. These qualities have been evaluated to a moderate extent in *J. cinerea*, *J. ailantifolia*, and their hybrids. Crystal and Jacobs (2014) reported that the hybrids exhibited both intermediate drought and flood tolerance relative to their *J. cinerea* (more drought tolerant) and *J. ailantifolia* (more flood tolerant) progenitors. Phenotypically, Crystal et al. (2016) projected that most hybrids will tend more toward their *J. ailantifolia* progenitor, although some hybrids did occupy the same space as their *J. cinerea* progenitor. The concerns of dissimilar hybrid and *J. cinerea* phenotypes, along with the intermediate environmental tolerances of the hybrids, could limit their ability to act as a suitable replacement for *J. cinerea*, potentially changing the distribution of the species. However, in a phenotypical study of *C. dentata* hybrids and their progenitors, which are at a much more advanced breeding stage than *J. cinerea* hybrids, 96% of hybrid trees in the third backcross generation were distinctly different from their *C. mollissima* progenitor, and closely resembled *C. dentata* (Diskin et al., 2006). Thus, using *C. dentata* as an example threatened hardwood species for restoration (Jacobs et al., 2013); it may be possible to develop hybrids that are similar to *J. cinerea*, at least phenotypically, with careful selection and breeding.

### Conclusions

Differences in canker occurrence and size by *Ocj* isolates were observed in this study, which may explain some of the differences in BCD severity reported among different *J. cinerea* populations. Hybrid families had smaller and fewer cankers and greater genetic gains compared to *J. cinerea* families, demonstrating that hybrids could be a possible breeding tool for developing BCD-resistant *J. cinerea* trees. Further, the genetic gain separation of families by canker size indicates potential heritability of BCD tolerance (under the timeframe of the current study). This is promising for the development of resistance breeding programs using hybrids, but possibly *J. cinerea* as well. Hybridization in *J. cinerea* is one of just a few examples in plants where hybrids are being considered not only for preserving a species’ economic value (timber and nut production), but also for ecological (restoration and conservation) and cultural purposes (ethnobotanical and medicinal). Thus, this study provides further evidence that hybrids represent a potentially effective tool for incorporating disease resistance to aid in restoration of threatened tree species.

## Data Availability Statement

The raw data supporting the conclusions of this article will be made available by the authors, without undue reservation.

## Author Contributions

AB analyzed the data, interpreted the results, and wrote the original manuscript. JM designed and executed the experiment, contributed to interpretation of the results, and revised the manuscript. SH and DJ contributed to interpretation of the results and revised the manuscript. All authors contributed to the article and approved the submitted version.

### Conflict of Interest

The authors declare that the research was conducted in the absence of any commercial or financial relationships that could be construed as a potential conflict of interest.
