# Supplementary material for: Hybrid Breeding for Restoration of Threatened Forest Trees: Evidence for Incorporating Disease Tolerance in Juglans cinerea
Source: Front Plant Sci. 2020 Oct 16;11:580693. doi: 10.3389/fpls.2020.580693 (PMC7596304; doi:10.3389/fpls.2020.580693)
Supplement: Supplementary file 1 [file Table_1.docx]

***Supplementary Material***

Table S1. Plant material used in the butternut canker disease tolerance screening by species or hybrid (*Juglans cinerea* or *J. cinerea* × *J. ailantifolia*), family (accession number assigned to each family by the Hardwood Tree Improvement and Regeneration Center), origin (location where the original mother tree scion or seed material was collected from), and number of trees.

| Species/Hybrid | Family | Origin | Trees (no.) |
| --- | --- | --- | --- |
| *J. cinerea* | 709 | Caledonia, MN | 10 |
| *J. cinerea* | 712 | Arlington, WI | 8 |
| *J. cinerea* | 713 | Rochester, MN | 8 |
| *J. cinerea* | 714 | Rochester, MN | 9 |
| *J. cinerea* | 715 | Rochester, MN | 6 |
| *J. cinerea* | 716 | Rochester, MN | 9 |
| *J. cinerea* | 717 | Whitewater, WI | 8 |
| *J. cinerea* | 718 | Whitewater, WI | 10 |
| *J. cinerea* | 722 | Nicolet NF, WI | 10 |
| *J. cinerea* | 723 | Whitewater, WI | 8 |
| *J. cinerea* | 726 | Mazaska Lake, MN | 8 |
| *J. cinerea* | 727 | Rochester, MN | 9 |
| *J. cinerea* | 728 | M. Twain NF,MO | 7 |
| *J. cinerea* | 730 | M. Twain NF,MO | 10 |
| *J. cinerea* | 733 | Perch River, NY | 10 |
| *J. cinerea* | 736 | Berlin, VT | 9 |
| *J. cinerea* | 738 | Trade Lake, WI | 8 |
| *J. cinerea* | 741 | Whitewater, WI | 10 |
| *J. cinerea* | 742 | Stratford, NH | 8 |
| *J. cinerea* | 743 | 'Creighton', PA | 9 |
| *J. cinerea* | 744 | 'Painter', IA | 9 |
| *J. cinerea* | 746 | Whitewater, WI | 10 |
| *J. cinerea* | 747 | Bark River, MI | 10 |
| Hybrid | 702 | New Paris, IN | 6 |
| Hybrid | 704 | Plymouth, IN | 9 |
| Hybrid | 706 | New Paris, IN | 3 |
| Hybrid | 707 | Brimfield, IN | 7 |
| Hybrid | 708 | Steuben Co., IN | 7 |
| Hybrid | 710 | Madison, WI | 10 |
| Hybrid | 711 | Madison, WI | 9 |
| Hybrid | 731 | Clover Lick, WV | 11 |
| Hybrid | 732 | Loudon, NH | 9 |
| Hybrid | 734 | Sanford, ME | 9 |
| Hybrid | 735 | Sanford, ME | 10 |
| Hybrid | 748 | Chequam NF, WI | 6 |
| Hybrid | 750 | Ankeny, IA | 10 |
